# Do NGS‐based techniques represent a first‐line testing in suspected Duchenne muscular dystrophy?

**DOI:** 10.1002/ccr3.5916

**Published:** 2022-06-02

**Authors:** Seyed Mohammad Hosseini, Nosratollah Alizadeh, Abolfazl Amini, Javad Mohammadi‐Asl

**Affiliations:** ^1^ Laboratory Sciences Research Center Golestan University of Medical Sciences Gorgan Iran; ^2^ Alizadeh Medical Genetic Counseling Center Abdanan Iran; ^3^ Department of Medical Biotechnology Faculty of Advanced Technologies in Medicine Golestan University of Medical Sciences Gorgan Iran

**Keywords:** Duchenne Muscular Dystrophy, genetic diagnosis, molecular diagnosis, next‐generation sequencing

## Abstract

Duchenne muscular dystrophy (DMD) is caused by mutations in the dystrophin gene, which mostly affects boys. The subject was an 8‐year‐old child who had typical symptoms of muscle weakness. The NGS may be used as an efficient and cost‐effective molecular diagnostic strategy for identifying patients with DMD.

## Graphical abstract

Duchenne muscular dystrophy (DMD) is one of the most common progressive neuromuscular diseases that caused by a mutation in the dystrophin gene. The subject was an 8‐year‐old child who had typical symptoms of muscle weakness. In this article, we suggest that The NGS may be used as an efficient and cost‐effective molecular diagnostic strategy for identify patients with DMD.

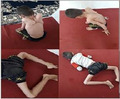

## 1 BACKGROUND

Myopathies are skeletal muscle disorders that commonly cause proximal muscle weakness.^1^ Duchenne muscular dystrophy is a severe form of muscular dystrophy caused by a mutation in the dystrophin gene (DMD; OMIM: 310200), and a milder form, Becker muscular dystrophy (BMD; OMIM: 300376), which are the most common types of dystrophies.^2^ Duchenne muscular dystrophy is one of the most common progressive neuromuscular diseases with inherited sex‐linked inheritance that generally affects only boys, with an estimated incidence of 1/3600 male births worldwide, and females who are carriers have milder symptoms.^3^ The dystrophin gene is the largest described human gene, located at Xp21.2 and comprises 79 exons with more than 2.5 Mb span of DNA.^4^ This gene produces a protein located primarily in skeletal and cardiac muscle and small amounts in the brain.^5^ DMD is caused by different mutations, containing a deletion of one or more exons is responsible for approximately 60%–70% of mutations, duplications are responsible for 5–10% of cases, small mutations (small deletions or insertions, missense, and nonsense mutations and splicing mutations) are almost responsible for 25%–35% of mutations, and approximately 2% of the remaining mutations are due to intronic rearrangements.^6^ In healthy muscle, dystrophin protein plays an essential role in the stabilization of muscle fiber membranes and the connection of the extracellular basement membrane with the intracellular cytoskeleton.^7^ In the absence of dystrophin, the permeability of the cell membrane increases, allowing intracellular creatine kinase and intracellular calcium to enter the serum. Persistent inflammation is initially associated with necrosis and hypertrophy, followed by progressive loss of regeneration and muscle degeneration, which is characteristic of DMD.^8^ Duchenne muscular dystrophy is a rapidly progressing disease, and all patients usually need to use a wheelchair by age 10; DMD patients develop a severe type of cardiomyopathy, which generally appears at age 10, and most of them die due to heart diseases and respiratory disorders.^9^, ^10^ The average life expectancy in patients with Duchenne muscular dystrophy is between 20 and 30 years.^11^ As reported in studies, small mutations in the dystrophin gene are one of the causes of Duchenne muscular dystrophy, which is costly and inefficient to detect by traditional sequencing methods such as Sanger. The MLPA method is also used to identify large mutations.^12^ In this study, the NGS technique was used to sequence and identify micro‐duplications in the dystrophin gene.

The physical examination of the patient, as well as the results of muscle biopsy, increased creatine kinase (CK) enzyme, electromyography (EMG), and molecular tests for dystrophin gene analysis, are used to diagnose DMD.

## 2 CASE REPORT

### 2.1 Case history

**FIGURE 1:**
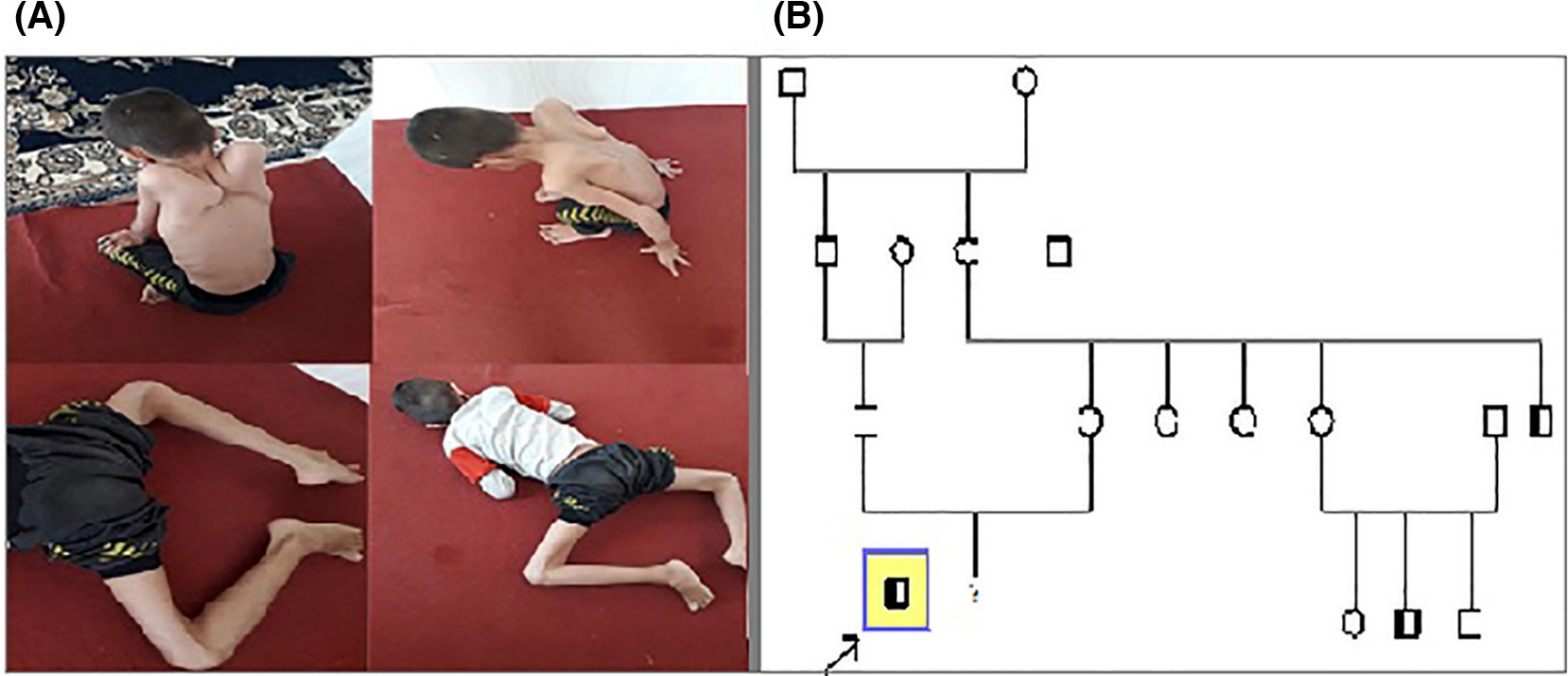
(A) Patient with DMD. (B) The pedigree of the study family; In the family tree, squares indicate male and circles indicate female of the family member. The arrow indicates the proband

In this case study, the subject of our study is an 8‐year‐old child with muscle weakness in his arms and legs and carried these symptoms from childhood. Walking difficulties and a chest deformity are two of the most common clinical issues seen. His parents are not consanguineous, and the child has a normal IQ. Muscle biopsy and histological examinations were not performed for him. The biochemical study showed abnormal high level of creatine phosphokinase (CPK) enzyme (13,369 U/L) and lactate dehydrogenase (LDH) enzyme (4544 U/L). Electromyogram (EMG) was performed and the electrical findings are consistent with a chronic myopathic pattern in proximal muscles. The subject has a positive family history. His maternal uncle died of muscular dystrophy, and his cousin also suffered from the disease (Figure 1).

### 2.2 Next‐generation sequencing (NGS)

The Multiplex ligation‐dependent probe amplification (MLPA) technique is commonly used as the first line of molecular detection, but because the proband's genome had no large deletions or duplications, NGS was used as the next step in the diagnosis. The proband genome sequence was performed using a custom‐designed Nimblegen chip capturing the dystrophin gene followed by next‐generation sequencing. The test platform examined >95% of the target gene, with over 99% sensitivity. Point mutation, micro‐insertion, deletion, and duplication could be simultaneously detected. The results of this sequencing were analyzed by bioinformatics methods with international databases of mutations and polymorphisms. The entire NGS procedure was performed by Macrogen Inc.

### 2.3 Multiplex ligation‐dependent probe amplification (MLPA)

**FIGURE 2:**
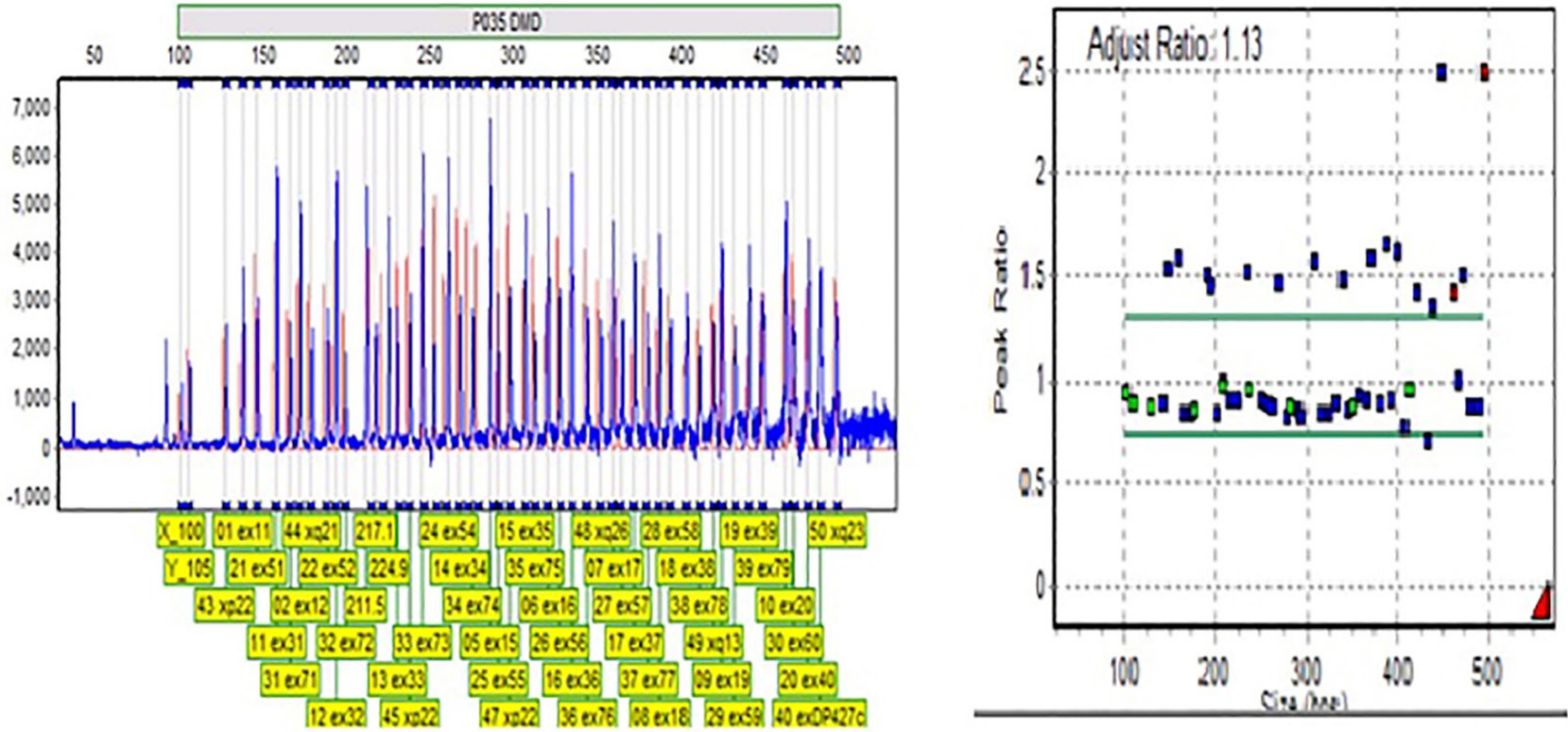
Multiplex ligation‐dependent probe amplification (MLPA) analysis of proband shows duplications DMD from exon 10 to exon 42

Seventy‐nine exons of the *DMD* gene were analyzed by the MLPA technique using SALSA MLPA KIT P034‐B2/P035‐B1 (MRC Holland BV) to detect exon deletions or duplications. All steps of this test are performed according to the manufacturer's instructions on the DNA extracted from the patient's blood (Figure 2).

## 3 RESULTS

Sequencing of this individual genome was performed, and the data were analyzed to identify previously reported and novel variants in different genes implicated in myotonic dystrophy. The results showed that no strict pathogenic point mutation was found by exome sequencing. The results of our data showed micro‐duplications in exons 10 to 42 of the dystrophin gene. The patient's DNA sample was investigated for the deletion and duplication in the Dystrophin gene for all 79 exons by the MLPA method. MLPA testing has confirmed these micro‐duplications. Molecular analysis of detected DMD disease duplication using MLPA and linkage in fetal sample's dystrophin gene revealed that the fetus was female and thus not affected by this family's pregnant mother.

## 4 DISCUSSION

Small mutations, rather than the large deletions and duplications seen in DMD, should be considered accurate molecular detection. Molecular DMD diagnosis is critical for identifying women who have a carrier in their family, which is important because it allows affected individuals to be identified in future pregnancies and prevented from being born. Only large deletion mutations and duplications in the dystrophin gene can be detected using the MLPA technique. Small mutations that could cause DMD disease are still unknown. The whole dystrophin gene must be sequenced to detect small mutations, which is time‐consuming and costly. The NGS technique has recently emerged as a method for detecting small mutations and duplications in order to identify all regions of the DMD gene. Small mutations, rather than the large deletions and duplications seen in DMD, should be considered accurate molecular detection. Molecular DMD diagnosis is critical for identifying women who have a carrier in their family, which is important because it allows affected individuals to be identified in future pregnancies and prevented from being born. Only large deletion mutations and duplications in the dystrophin gene can be detected using the MLPA technique. Small mutations that could cause DMD disease are still unknown. The whole dystrophin gene must be sequenced to detect small mutations, which is time‐consuming and costly. The NGS technique has recently emerged as a method for detecting small mutations and duplications in order to identify all regions of the DMD gene. Analyzing the type and frequency of 7149 DMD mutations indicated that among 5682 large mutations (80% of mutations), 86% were deletions, and 14% were duplications. There were 1445 small mutations (20% of mutations), included were 25% deletions, 9% duplications, 14% splice site mutations, and 52% point mutations.^13^

In the present study, we detected micro‐duplications in exons 10 to 42 by NGS technique, and then the results were confirmed by the MLPA technique. Our results provide evidence that the NGS technique can be used in dystrophin gene sequencing to diagnose patients with DMD.

## 5 CONCLUSION

Because DMD is a serious illness for which there is currently no effective treatment, it is critical to avoid the birth of affected male children. Identifying female carriers through genetic counseling and molecular testing can help prevent the disease. In this paper, we propose that the NGS technique may be used as an efficient and cost‐effective molecular diagnostic strategy to identify micro‐duplication mutations in patients with Duchenne muscular dystrophy, which are difficult and expensive with traditional methods. More research is needed in the future to review and confirm the findings of our study.

## AUTHOR CONTRIBUTIONS

SMH and JMA prepared the first draft of the manuscript and take part in the management of the patient. NA performed an analysis of literature in this field and improved a draft. AA involved in taking medical care about patient. All authors were involved in the writing, revision, and final review of the manuscript.

## CONFLICT OF INTEREST

The authors declare no conflict of interest.

## ETHICAL APPROVAL

This case report has observed the ethical guidelines.

## CONSENT

Patient consent was obtained for all data collected for this study, with written informed consent obtained from the patient to publish this report in accordance with the journal's patient consent policy.

## Data Availability

The data that support the findings of this study are available from the corresponding author upon reasonable request.
